# The Effects of Alpha Interferon on the Development of Autoimmune Thyroiditis in the NOD H2h4 Mouse

**DOI:** 10.1080/10446670310001642177

**Published:** 2003

**Authors:** Yael Oppenheim, Grace Kim, Yoshiyuki Ban, Pamela Unger, Erlinda Concepcion, Takao Ando, Yaron Tomer

**Affiliations:** 1 Division of Endocrinology, Diabetes and Bone Diseases New York NY USA; 2 Department of Pathology Mount Sinai School of Medicine New York NY USA

## Abstract

Alpha interferon (αIFN) therapy is known to induce thyroid autoimmunity in up to 40% of patients. The mechanism is unknown, but Th1 switching has been hypothesized. The aim of our study was to examine whether αIFN accelerated the development of thyroiditis in genetically susceptible mice. We took advantage of NOD-H2h4, a genetically susceptible animal model, which develops thyroiditis when fed a high iodine diet. Six to eight week old male NOD H2h4 mice were injected with mouse αIFN (200 units) or with saline three times a week for 8 weeks. All mice drank iodinated water (0.15%). Mice were sacrificed after 8 weeks of injection. Their thyroids were examined for histology and blood was tested for antithyroglobulin antibody levels. T4 and glucose levels were also assessed. In the IFN-injected group, 6/13 (46.2%) developed thyroiditis and/or thyroid antibodies while in the saline-injected group, only 4/13 (30.8%) developed thyroiditis and/or thyroid antibodies (*p*=0.4). The grade of thyroiditis was not different amongst the two groups. None of the mice developed clinical thyroiditis or diabetes mellitus. Our results showed that αIFN treatment did not accelerate thyroiditis in this mouse model. This may imply that αIFN induces thyroiditis in a non-genetically dependent manner, and this would not be detected in a genetically susceptible mouse model if the effect were small. Alternatively, it is possible that αIFN did not induce thyroiditis in mice because, unlike in humans, in mice αIFN does not induce Th1 switching.


*Clinical & Developmental Immunology*, June–December 2003 Vol. 10 (2–4), pp. 161–165

Taylor & Francis healthsciences

### The Effects of Alpha Interferon on the Development of Autoimmune Thyroiditis in the NOD H2h4 Mouse

YAEL OPPENHEIM[a,*], GRACE KIM[a,*], YOSHIYUKI BAN[a,*], PAMELA UNGER[b], ERLINDA CONCEPCION[a], TAKAO ANDO[a] and YARON TOMER[a,†]

[a]*Division of Endocrinology, Diabetes, and Bone Diseases, New York, NY, USA;* [b]*Department of Pathology, Mount Sinai School of Medicine, New York, NY, USA*

Alpha interferon (αIFN) therapy is known to induce thyroid autoimmunity in up to 40% of patients. The mechanism is unknown, but Th1 switching has been hypothesized. The aim of our study was to examine whether αIFN accelerated the development of thyroiditis in genetically susceptible mice. We took advantage of NOD-H2h4, a genetically susceptible animal model, which develops thyroiditis when fed a high iodine diet. Six to eight week old male NOD H2h4 mice were injected with mouse αIFN (200 units) or with saline three times a week for 8 weeks. All mice drank iodinated water (0.15%). Mice were sacrificed after 8 weeks of injection. Their thyroids were examined for histology and blood was tested for antithyroglobulin antibody levels. T4 and glucose levels were also assessed. In the IFN-injected group, 6/13 (46.2%) developed thyroiditis and/or thyroid antibodies while in the saline-injected group, only 4/13 (30.8%) developed thyroiditis and/or thyroid antibodies ($p = 0.4$). The grade of thyroiditis was not different amongst the two groups. None of the mice developed clinical thyroiditis or diabetes mellitus. Our results showed that αIFN treatment did not accelerate thyroiditis in this mouse model. This may imply that αIFN induces thyroiditis in a non-genetically dependent manner, and this would not be detected in a genetically susceptible mouse model if the effect were small. Alternatively, it is possible that αIFN did not induce thyroiditis in mice because, unlike in humans, in mice αIFN does not induce Th1 switching.

*Keywords*: Alpha interferon; Autoimmunity; Animal model; Thyroiditis

### INTRODUCTION

Alpha interferon (αIFN) treatment for various medical conditions is known to induce thyroid autoimmunity in up to 40% of patients. The association between αIFN treatment and autoimmune thyroiditis was reported as early as 1986 by Burman *et al.* (1986), who studied patients receiving αIFN for carcinoid tumors. The association is most recognized in patients with hepatitis C (Watanabe *et al.*, 1994), although it has been noted to occur in several other conditions treated by αIFN including breast cancer, carcinoid and hematologic malignancies (Fentiman *et al.*, 1988; Ronnblom *et al.*, 1991a,b; Gisslinger *et al.*, 1992; Silvestri *et al.*, 1994; Watanabe *et al.*, 1994). The thyroid diseases associated with αIFN therapy include both autoimmune hyperthyroidism and hypothyroidism (Koh *et al.*, 1997). In up to 40% of cases, thyroid dysfunction persists after discontinuation of the αIFN therapy (Koh *et al.*, 1997).

Factors that predispose patients to the development of αIFN-induced thyroiditis include hepatitis C infection, positive pre-treatment antithyroid antibodies, dose and duration of αIFN therapy, genetic factors, stress, dietary iodine intake, female gender, pregnancy and HLA-A2 in the Japanese population (Preziati *et al.*, 1995; Roti *et al.*, 1996; Custro *et al.*, 1997; Koh *et al.*, 1997; Minelli *et al.*, 1997; Fernandez-Soto *et al.*, 1998; Dumoulin *et al.*, 1999; Kakizaki *et al.*, 1999, 2000). The mechanisms by which αIFN induces thyroid autoimmunity are unclear.

We hypothesized that αIFN accelerates subclinical thyroiditis by immune modulation in genetically predisposed individuals. In order to examine this hypothesis, we took advantage of the NOD H2h4 mouse model, a model in which the mice are genetically prone to develop

*These authors contributed equally to the work presented in this manuscript.

†Corresponding author. Address: Division of Endocrinology, Diabetes, and Bone Diseases, Box 1055, Mount Sinai School of Medicine, One Gustave L. Levy Place, New York, NY, USA. Tel.: +1-212-241-7085. Fax: +1-212-241-4218. E-mail: yaron.tomer@mssm.edu

ISSN 1740-2522 print/ISSN 1740-2530 online © 2003 Taylor & Francis Ltd
DOI: 10.1080/10446670310001642177

thyroiditis when placed on iodinated drinking water (Rasooly *et al.*, 1996; Braley-Mullen *et al.*, 1999). We used the NOD H2h4 mouse model to examine the effects of αIFN on animals genetically susceptible to thyroiditis.

### MATERIALS AND METHODS

### Animal Protocol

Mouse interferon alpha was obtained from PBL Biomedical Laboratories (New Brunswick, NJ). Six to eight week old NOD H2h4 male mice were purchased from Taconic Farms (Germantown, NY). Male mice were used in our experiments since previous studies (Rasooly *et al.*, 1996; Braley-Mullen *et al.*, 1999) and our own experiments revealed that male mice are more prone to thyroiditis in this model (data not shown). Thirteen mice were injected intra peritoneally (i.p.) with αIFN (200 units, 0.4 cc) three times a week for eight weeks. As controls, 13 mice were injected with saline (0.4 cc) three times a week for eight weeks. After eight weeks of injections, mice were sacrificed, 1 ml of blood was drawn, and their thyroids were removed. All these 26 mice were given 0.15% iodinated drinking water [Sodium Iodide, Sigma Chemical Company (St. Louis, MO)]. Additional 14 mice (6 injected with saline and 8 injected with αIFN) received regular water without iodine supplementation.

### Measurement of Murine Tg (mTg) Antibodies

Serum was analyzed for mTg antibodies by an ELISA assay as previously described (Imaizumi *et al.*, 2001). Briefly, 96 well-plates (Immulon 1, Dynex Technologies Inc., Pittsburgh, PA) were coated with mouse thyroglobulin (0.1 mcg thyroglobulin/well diluted in Carbonate/Bicarbonate Buffer, pH 9.6) and incubated overnight at 4°C. The plates were washed with 0.05% PBS-Tween and nonspecific binding was blocked for 30 min with 3% BSA-PBS/Tween. After washing, the sera were diluted 1:100 in 0.5% BSA/PBS and added to the plates for 2 h at room temperature. Plates were washed again 6 times and alkaline phosphatase conjugated anti mouse IgG (Sigma Diagnostics, St. Louis, MO) was added for 30 min. Plates were washed 4 times and then developed using p-nitrophenyl phosphate as substrate. Absorbance was read at 405 nm. Samples were considered positive if the OD was 2 standard deviation (SD) above the average OD for controls.

### Thyroid Histology

Mice thyroids were fixed in 10% formalin and stained by hematoxylin and eosin. The severity of thyroiditis was graded on a scale from +1 to +4.0 as follows: +1, small focal areas of lymphocytic cells; +2, focal collection of lymphocytic cells with some follicular destruction; +3, diffuse lymphocytic infiltration of thyroid follicles

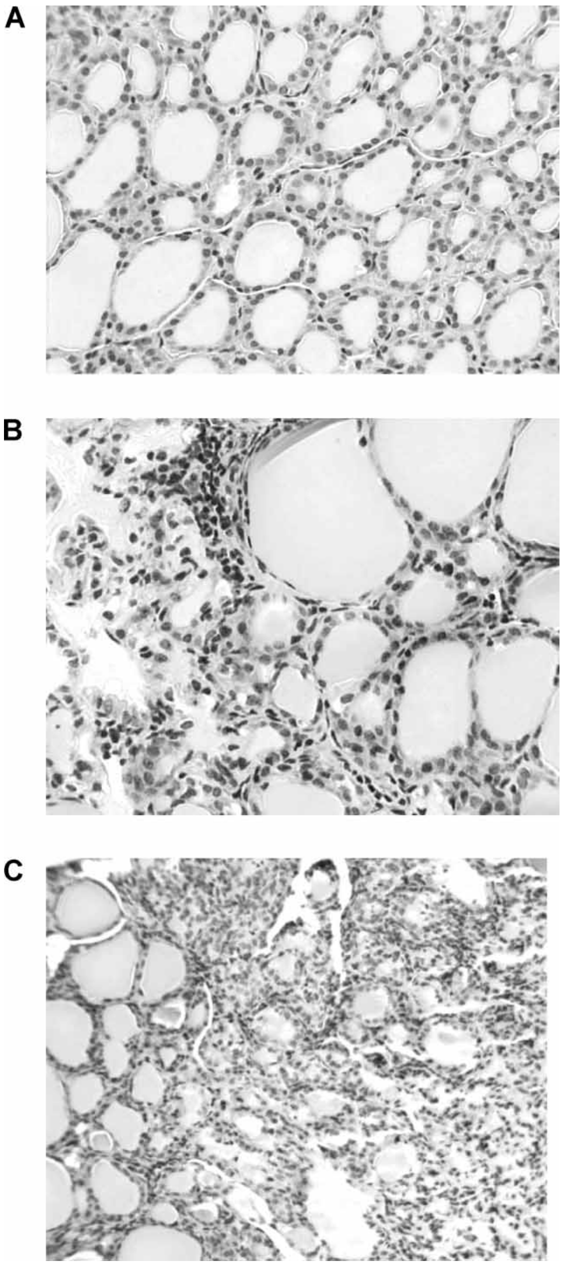

FIGURE 1 Histological grading of thyroiditis. After eight weeks injections, mice were sacrificed. Thyroids were removed and fixed and stained by H&E. (A) Normal mouse thyroid, (B) Grade 1 thyroiditis, (C) Grade 2 thyroiditis. None of our mice developed thyroiditis with severity > +2.

involving approximately 40% or less of the thyroid and +4, >40% lymphocytic infiltration of thyroid follicles (Fig. 1; Imaizumi *et al.*, 2001).

### Measurement of Total Thyroxine (T4) and Glucose Levels

T4 was measured by radioimmunoassay using the neonatal T4 Coat-A-Count kit (Diagnostics Products Corporation, Los Angeles, CA). Glucose levels were measured using the One Touch Profile Glucometer (Lifescan Inc., Milpitas, CA).

### Data Analysis

Data were analyzed using Student's, Chi-Square and Fisher Exact tests. Probability values less than 0.05 were considered significant.

### RESULTS

### Effects of Iodine on Development of Thyroiditis

None of the 14 mice that did not receive iodine supplementation (6 injected with saline and 8 injected with αIFN) developed thyroiditis. In contrast, 10/26 (38.5%) of the mice that received iodine supplementation developed thyroiditis ($p = 0.007$).

### Autoantibody Levels in αIFN-injected Mice and Controls

Positive thyroglobulin antibodies were detected in 3/13 (23%) of the mice injected with αIFN compared to 0% of the control mice injected with saline ($p = 0.07$).

### Thyroid Histology in αIFN-injected Mice and Controls

Thyroid infiltration was detected in 5/13 (38.5%) of the αIFN-injected mice and 4/13 (30.8%) in the control mice (Table I). The grade of thyroiditis was not different between the αIFN-injected versus controls. When we used positive thyroid antibodies and/or positive histology to define thyroiditis, 6/13 (46.2%) of the mice injected with αIFN developed thyroiditis compared to 4/13 (30.8%) in the control mice (Table I). This difference did not reach statistical significance ($p = 0.4$).

### Clinical Disease in αIFN-injected Mice and Controls

None of the mice developed evidence of clinical thyroid dysfunction and/or diabetes mellitus. There were no significant differences in the T4 levels between the 4 groups mice (Fig. 2). Moreover, the levels of T4 were similar in the mice that developed thyroiditis and those that did not (average Total T4 level = 4.24 in mice that developed thyroiditis vs. 4.19 in mice that did not develop thyroiditis) (Fig. 2).

Since it has been previously reported that insulitis develops in 20–30% of NODH2h4 mice

TABLE I Thyroiditis and thyroglobulin antibody frequency in mice injected with saline and αIFN

| | Saline injection ($n$=13) | αIFN injection ($n$=13) | $P$ value |
|---|---|---|---|
| Positive histology for thyroiditis | 4 (31%) | 5 (39%) | NS* |
| Thyroiditis on histology and/or elevated Tg-Ab's | 4 (31%) | 6 (46%) | NS |

* NS, not significant.

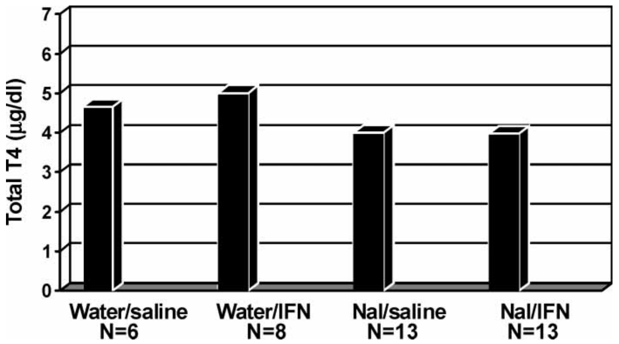

FIGURE 2 T4 levels in mice injected with IFN or saline and fed with water or NaI. There were no significant differences in the T4 levels of mice that developed thyroiditis and those that did not in both groups (average Total T4 level = 4.24 vs. 4.19).

(Braley-Mullen *et al.*, 1999), we also tested blood glucose levels. There were no significant differences in the glucose levels between mice that developed thyroiditis and those that did not in both groups (average glucose level = 174 vs. 165.2) (Fig. 3).

### DISCUSSION

Interferons are cytokines that are involved in immune modulation, possessing both antiviral and antitumoral activity. Interferons are grouped into Types I and II. Type I interferons are acid stable and include alpha, beta and omega subtypes. Type II inteferons are acid labile and include the gamma subtype (Walter *et al.*, 1998). αIFN first became available for use in humans in the form of leukocyte-derived interferon in the mid 1970's. Subsequently, in 1986, recombinant αIFN became available for clinical use. Since that time, recombinant interferons have been used to treat a wide spectrum of medical conditions, including hepatitis, and several cancers (Walter *et al.*, 1998).

Interferons were shown to induce and/or exacerbate several autoimmune diseases, including autoimmune thyroiditis, autoimmune hepatitis, rheumatoid and lupus like diseases, and autoimmune diabetes mellitus. Interestingly, thyroid disease is the most prevalent autoimmune disease exacerbated or induced by αIFN (Dumoulin *et al.*, 1999). It is unclear whether this reflects the higher prevalence of thyroid autoimmunity in the general population or a thyroid specific effect of interferon.

Our hypothesis was that αIFN accelerates thyroiditis in genetically prone individuals. Evidence supporting our hypothesis is that most of the risk factors for αIFNinduced thyroiditis are genetic (e.g. positive pre-treatment antithyroid antibodies, and female gender) (Preziati *et al.*, 1995; Roti *et al.*, 1996; Custro *et al.*, 1997; Minelli *et al.*, 1997; Tunbridge *et al.*, 1977; Koh *et al.*, 1997; Fernandez-Soto *et al.*, 1998; Kakizaki *et al.*, 1999; Dumoulin *et al.*, 1999; Kakizaki *et al.*, 2000). In order to

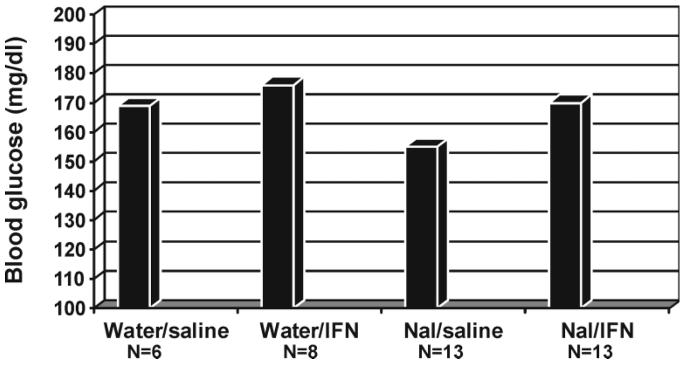

FIGURE 3 Glucose levels in mice injected with IFN or saline and fed with water or NaI. There were no significant differences in the glucose levels between mice that developed thyroiditis and those that did not in both groups (average glucose level = 174 vs. 165.2).

test our hypothesis, we gave αIFN to mice genetically susceptible to thyroiditis.

Our study revealed that αIFN did not accelerate thyroiditis in this thyroiditis-prone mouse model. Thus our study does not support an accelerating effect of αIFN on autoimmune thyroid disease development at least in our mouse model. Therefore, it is possible that αIFN induces thyroiditis de novo and does not accelerate disease development in genetically predisposed individuals. Such a de novo effect would not be detected in our mice, which are already prone for thyroiditis, especially if this is a mild effect. Alternatively, it is possible that αIFN did accelerate thyroiditis development in the NOD H2h4 mice but that the effect was too small to be detected in our experiments. Our studies showed a higher frequency of thyroiditis in the αIFN injected group versus the controls (48.2% and 30.8%, respectively), however this difference did not reach statistical significance. Power calculations show that at least 80 mice per group would have been required to reach statistical significance for the difference that we observed. Additionally, there is some variability in the incidence of thyroiditis in this mouse model, which may have interfered with detection of a small effect (Rasooly *et al.*, 1996; Braley-Mullen *et al.*, 1999). Another explanation for the lack of an effect of αIFN in the mice may be due to its inability to induce Th1 switching in mice. Unlike in humans, αIFN does not induce Th1 switching in mice because of its failure to activate the signal transducer and activator of transcription 4 (Stat4) (Farrar and Murphy, 2000). Some authors have suggested that αIFN accelerates human thyroiditis by inducing Th1 switching (Farrar and Murphy, 2000) and, if this is indeed the case, the lack of αIFN-induced thyroiditis in our mice may reflect the absence of αIFN-induced Th1 differentiation and activation of Stat4 in mice. However, it is unclear whether Th1 switching can explain αIFN-induced thyroiditis as 40% of patients develop Graves' disease, generally believed to be a Th2 mediated disease (Davies, 2000).

In summary, αIFN did not accelerate thyroiditis in this mouse model. However, minor αIFN effects cannot be excluded. Additional studies are needed to examine this possibility.

### *Acknowledgements*

We thank Dr Terry F Davies for his teaching, support and ever ready help in our joint studies. This work was supported in part by grants DK61659 & DK58072 from NIDDKD (to YT).

### *References*

Braley-Mullen, H., Sharp, G.C., Medling, B. and Tang, H. (1999) "Spontaneous autoimmune thyroiditis in NOD.H-2h4 mice", *J. Autoimmun.* **12**, 157–165.

Burman, P., Totterman, T.H., Oberg, K. and Karlsson, F.A. (1986) "Thyroid autoimmunity in patients on long term therapy with leukocyte-derived interferon", *J. Clin. Endocrinol. Metab.* **63**, 1086–1090.

Custro, N., Montalto, G., Scafidi, V., et al. (1997) "Prospective study on thyroid autoimmunity and dysfunction related to chronic hepatitis C and interferon therapy", *J. Endocrinol. Invest.* **20**, 374–380.

Davies, T.F. (2000) "Graves' disease: pathigenesis", In: Braverman, L.E. and Utiger, R.D., eds, Werner and Ingbar's the Thyroid: A Fundamental and Clinical Text (Lippincott, Williams & Wilkens, Philadelphia), pp 518–530.

Dumoulin, F.L., Leifeld, L., Sauerbruch, T. and Spengler, U. (1999) "Autoimmunity induced by interferon-alpha therapy for chronic viral hepatitis", *Biomed. Pharmacother.* **53**, 242–254.

Farrar, J.D. and Murphy, K.M. (2000) "Type I interferons and T helper development", *Immunol. Today* **21**, 484–489.

Fentiman, I.S., Balkwill, F.R., Thomas, B.S., Russell, M.J., Todd, I. and Bottazzo, G.F. (1988) "An autoimmune aetiology for hypothyroidism following interferon therapy for breast cancer", *Eur. J. Cancer Clin. Oncol.* **24**, 1299–1303.

Fernandez-Soto, L., Gonzalez, A., Escobar-Jimenez, F., *et al.* (1998) "Increased risk of autoimmune thyroid disease in hepatitis C vs hepatitis B before, during, and after discontinuing interferon therapy", *Arch. Intern. Med.* **158**, 1445–1448.

Gisslinger, H., Gilly, B., Woloszczuk, W., *et al.* (1992) "Thyroid autoimmunity and hypothyroidism during long-term treatment with recombinant interferon-alpha", *Clin. Exp. Immunol.* **90**, 363–367.

Imaizumi, M., Pritsker, A., Kita, M., Ahmad, L., Unger, P. and Davies, T.F. (2001) "Non-MHC driven exacerbation of experimental thyroiditis in the postpartum period", *Autoimmunity* **34**, 95–105.

Kakizaki, S., Takagi, H., Murakami, M., Takayama, H. and Mori, M. (1999) "HLA antigens in patients with interferon-alpha-induced autoimmune thyroid disorders in chronic hepatitis C", *J. Hepatol.* **30**, 794–800.

Kakizaki, S., Takagi, H., Ichikawa, T., *et al.* (2000) "Histological change after interferon therapy in chronic hepatitis C in view of iron deposition in the liver", *Biol. Trace Elem. Res.* **73**, 151–162.

Koh, L.K., Greenspan, F.S. and Yeo, P.P. (1997) "Interferon-alpha induced thyroid dysfunction: three clinical presentations and a review of the literature", *Thyroid* **7**, 891–896.

Minelli, R., Braverman, L.E., Giuberti, T., *et al.* (1997) "Effects of excess iodine administration on thyroid function in euthyroid patients with a previous episode of thyroid dysfunction induced by interferon-alpha treatment", *Clin. Endocrinol. (Oxf)* **47**, 357–361.

Preziati, D., La Rosa, L., Covini, G., *et al.* (1995) "Autoimmunity and thyroid function in patients with chronic active hepatitis treated with recombinant interferon alpha-2a", *Eur. J. Endocrinol.* **132**, 587–593.

Rasooly, L., Burek, C.L. and Rose, N.R. (1996) "Iodine-induced autoimmune thyroiditis in NOD-H-2h4 mice", *Clin. Immunol. Immunopathol.* **81**, 287–292.

Ronnblom, L.E., Alm, G.V. and Oberg, K. (1991a) "Autoimmune phenomena in patients with malignant carcinoid tumors during interferon-alpha treatment", *Acta Oncol.* **30**, 537–540.

Ronnblom, L.E., Alm, G.V. and Oberg, K.E. (1991b) "Autoimmunity after alpha-interferon therapy for malignant carcinoid tumors", *Ann. Intern. Med.* **115**, 178–183.

Roti, E., Minelli, R., Giuberti, T., *et al.* (1996) "Multiple changes in thyroid function in patients with chronic active HCV hepatitis treated with recombinant interferon-alpha", *Am. J. Med.* **101**, 482–487.

Silvestri, F., Virgolini, L., Mazzolini, A., *et al.* (1994) "Development of autoimmune thyroid diseases during long-term treatment of hematological malignancies with alpha-interferons", *Haematologica* **79**, 367–370.

Tunbridge, W.M., Evered, D.C., Hall, R., *et al.* (1977) "The spectrum of thyroid disease in a community: the Whickham survey", *Clin. Endocrinol. (Oxf)* **7**, 481–493.

Walter, M.R., Bordens, R., Nagabhushan, T.L., *et al.* (1998) "Review of recent developments in the molecular characterization of recombinant alfa interferons on the 40th anniversary of the discovery of interferon", *Cancer Biother. Radiopharm.* **13**, 143–154.

Watanabe, U., Hashimoto, E., Hisamitsu, T., Obata, H. and Hayashi, N. (1994) "The risk factor for development of thyroid disease during interferon-alpha therapy for chronic hepatitis C", *Am. J. Gastroenterol.* **89**, 399–403.

